# Differential Expression of *Paraburkholderia phymatum* Type VI Secretion Systems (T6SS) Suggests a Role of T6SS-b in Early Symbiotic Interaction

**DOI:** 10.3389/fpls.2021.699590

**Published:** 2021-07-28

**Authors:** Sebastian Hug, Yilei Liu, Benjamin Heiniger, Aurélien Bailly, Christian H. Ahrens, Leo Eberl, Gabriella Pessi

**Affiliations:** ^1^Department of Plant and Microbial Biology, University of Zurich, Zurich, Switzerland; ^2^Agroscope, Research Group Molecular Diagnostics, Genomics and Bioinformatics, Swiss Institute of Bioinformatics, Wädenswil, Switzerland

**Keywords:** root nodule, legume, rhizobium, T6SS, competition, temperature regulation, citrate, C4-dicarboxylates

## Abstract

*Paraburkholderia phymatum* STM815, a rhizobial strain of the *Burkholderiaceae* family, is able to nodulate a broad range of legumes including the agriculturally important *Phaseolus vulgaris* (common bean). *P. phymatum* harbors two type VI Secretion Systems (T6SS-b and T6SS-3) in its genome that contribute to its high interbacterial competitiveness *in vitro* and in infecting the roots of several legumes. In this study, we show that *P. phymatum* T6SS-b is found in the genomes of several soil-dwelling plant symbionts and that its expression is induced by the presence of citrate and is higher at 20/28°C compared to 37°C. Conversely, T6SS-3 shows homologies to T6SS clusters found in several pathogenic *Burkholderia* strains, is more prominently expressed with succinate during stationary phase and at 37°C. In addition, T6SS-b expression was activated in the presence of germinated seeds as well as in *P. vulgaris* and *Mimosa pudica* root nodules. Phenotypic analysis of selected deletion mutant strains suggested a role of T6SS-b in motility but not at later stages of the interaction with legumes. In contrast, the T6SS-3 mutant was not affected in any of the free-living and symbiotic phenotypes examined. Thus, *P. phymatum* T6SS-b is potentially important for the early infection step in the symbiosis with legumes.

## Introduction

Crop production is often limited by nitrogen supply, even though nitrogen gas (N_2_) makes up 78% of the Earth’s atmosphere (Fields, 2004). A specialized group of prokaryotes, the diazotrophic bacteria, is able to convert inert atmospheric nitrogen into biologically available ammonium (NH_4_) by a process called biological N_2_ fixation (BNF), which plays an important role for sustainable food production by contributing up to 65% of nitrogen used in agriculture (Vance and Graham, 1995; Udvardi and Poole, 2013; Lindström and Mousavi, 2020). BNF by rhizobia involves the establishment of a symbiotic relationship, which leads to the formation of a specialized plant organ on the root or on the stem, the nodule (Masson-Boivin et al., 2009; Oldroyd et al., 2011; Wheatley et al., 2020). Upon sensing plant signal molecules produced by the roots (flavonoids), rhizobia produce lipochitooligosaccharides, called Nod factors (NFs), which modulate the growth of the root tip and induce root hair curling (Gage and Margolin, 2000; Cooper, 2007). A rhizobial microcolony grows in the curl and finally enters the root hair by hydrolysing the plant cell wall and inducing an invagination of the plasma membrane (Ibáñez et al., 2017). This invagination leads to the creation of a so called infection thread (IT) inside the root hair, in which the bacteria continue to grow (Gage and Margolin, 2000). The IT grows toward the base of root hair cells, ramifies within the root cortical tissue and releases the rhizobia within the cytoplasm of cortical cells via endocytosis, leading to an intracellular rhizobia-legume symbiosis. The rhizobia are then surrounded by a plant membrane forming an organelle like structure called a symbiosome. Within these symbiosomes, the bacteria differentiate into nitrogen-fixing bacteroids (Udvardi and Poole, 2013; Clarke et al., 2014; Ledermann et al., 2021). The rhizobial enzyme nitrogenase, a complex two-component metalloenzyme that consists of a homodimeric reductase (the Fe protein, encoded by *nifH*) and of a heterotetrameric dinitrogenase (the MoFe protein, encoded by *nifD* and *nifK*), reduces N_2_ into a plant usable nitrogen form (Halbleib and Ludden, 2000; Dixon and Kahn, 2004). Typical carbon sources delivered by the plant as energy source to the symbiont are C4-dicarboxylates such as succinate, malate and fumarate, which are transported by the dicarboxylate transporter A (DctA) inside bacterial cells (Janausch et al., 2002; Yurgel and Kahn, 2004). DctA is a secondary active symporter belonging to the glutamate transporter family and is known to be important for rhizobia to differentiate into nitrogen-fixing bacteroids (Ronson et al., 1981; Finan et al., 1983; Yurgel and Kahn, 2004). Thus, rhizobia are able to adapt to different lifestyles ranging from free-living growth in the soil to biofilm growth when colonizing the root, through stressful growth conditions inside the IT and finally as N_2_-fixing bacteroids inside the cells. Rhizobia are polyphyletic and include members of two classes of proteobacteria, the alpha-proteobacteria and since 2001 the beta-proteobacteria from the family *Burkholderiaceae* (Chen et al., 2001; Moulin et al., 2001; Masson-Boivin et al., 2009; Bontemps et al., 2010; Gyaneshwar et al., 2011). *Paraburkholderia phymatum* STM815, previously called *Burkholderia phymatum*, was isolated from root nodules in 2001 (Moulin et al., 2001, 2014; Gyaneshwar et al., 2011; Mishra et al., 2012) and was subsequently shown to have an exceptionally broad host range by nodulating over 40 different *Mimosa* species from South America, Asia, Africa and even papilionoid legumes such as the agriculturally important *Phaseolus vulgaris* (common bean) (Elliott et al., 2007; dos Reis et al., 2010; Talbi et al., 2010; Moulin et al., 2014; Lemaire et al., 2016). Competition experiments revealed that *P. phymatum* STM815 was more competitive than other alpha- and beta-rhizobia in infecting and nodulating the root of several legumes (Elliott et al., 2009; Melkonian et al., 2014; Lardi et al., 2017; Klonowska et al., 2018). This high competitiveness mainly depends on environmental conditions (nitrogen limitation as well as low pH favors *P. phymatum* dominance) and on host and symbiont genotype (Elliott et al., 2009; Garau et al., 2009). In other rhizobia, lipopolysaccharides (LPS), exopolysaccharides (EPS), antibiotic production (Bhagwat et al., 1991; Zdor and Pueppke, 1991; Robleto et al., 1998; Geddes et al., 2014), motility (Mellor et al., 1987), and catabolism of certain compounds (Kohler et al., 2010; Ding et al., 2012; Wheatley et al., 2020) have been shown to be important for high competitiveness. However, the factors that are important for competitiveness and promiscuity of *P. phymatum* are still largely unknown. However, in *P. phymatum* large genome two *P. phymatum* Type VI Secretion Systems (T6SSs) located on plasmid pBPHY01 (T6SS-b and T6SS-3) were shown to play a role in inter-bacterial competition (de Campos et al., 2017). The T6SS was first described in the human pathogens *Vibrio cholerae* and *Pseudomonas aeruginosa* and was later found in numerous other Gram-negative bacteria (Mougous et al., 2006; Pukatzki et al., 2006; Boyer et al., 2009). Interestingly, the T6SS was originally discovered in the rhizobium *Rhizobium leguminosarum* bv. *trifolii*, but not recognized as part of a secretion system at that time. The locus “*imp*” (for impaired in nodulation, now called *tssK*), was responsible for the impaired ability of this vetch-nodulating strain to infect pea (Roest et al., 1997; Bladergroen et al., 2003). The number of genes encoded within a T6SS cluster can vary between 16 and 38, although only 13 genes encode the core components needed for a fully functional T6SS (Boyer et al., 2009). The core system is composed of a membrane complex (TssJLM), a baseplate (TssAEFGK), a tail tube (TssD or Hcp), a tail tip (TssI or VgrG), and a contractile sheath (TssBC) (Cianfanelli et al., 2016). The membrane complex forms a channel through the cytoplasm membrane and the peptidoglycan layer and the baseplate is important for tube and sheath assembly (Zoued et al., 2014). The tube is composed of hexametric rings of hemolysin-coregulated proteins (Hcp) that assemble into a channel like structure and is covered by a valine-glycine repeat protein G (VgrG), which acts as a spike to penetrate the target cell. This VgrG can be topped again by proteins from the proline-alanine-alanine-arginine (PAAR) repeat superfamily to which effector proteins can be attached (Shneider et al., 2013). The purpose of the T6SS is to puncture and transport effector proteins into a eukaryotic or prokaryotic target cell. The T6SS effector proteins are often encoded within the T6SS gene cluster and usually located downstream of *vgrG* but can also be found outside of T6SS clusters, forming small clusters including effector and immunity genes (Zoued et al., 2014; Alcoforado Diniz et al., 2015; Santos et al., 2019). The effectors can be divided in three classes: cell wall degrading enzymes (e.g., muramidase and amidase), DNA or RNA targeting nucleases (e.g., AHH nuclease and pyocinS/colicin/Tde DNase), and membrane targeting (e.g., lipase) (Durand et al., 2014; Kapitein and Mogk, 2014; Ma et al., 2014; Salomon et al., 2014). These effectors can kill the target cells if the required immunity protein is missing (Dong et al., 2013; Yang et al., 2018). Only recently, a study reported for the first time a positive role for T6SS in rhizobial symbiosis. Three different *Rhizobium etli* Mim1 T6SS mutants deficient in *tssD*, *tssM* or lacking the whole structural gene cluster (*tssA – tagE*) led to the formation of smaller nodules in symbiosis with *P. vulgaris* (Salinero-Lanzarote et al., 2019). However, nitrogenase activity and symbiotic efficiency was not affected in nodules occupied by these mutants, potentially indicating that earlier steps of the symbiosis could be affected.

In this study, we first evaluated the phylogenetic conservation and expression of the two *P. phymatum* T6SS-b and T6SS-3 clusters *in vitro* in response to different carbon sources and temperatures. Furthermore, we explored the expression and role of the T6SS clusters during symbiosis, in the presence of germinated seeds and within root nodules. We show that T6SS-b is also present in other soil bacteria and its expression is elevated at lower temperature and in the presence of citrate as carbon source. In contrast, T6SS-3 is also found in pathogenic *Burkholderia* strains (*Burkholderia pseudomallei* and *Burkholderia mallei*), which may explain its higher expression at 37°C and in stationary phase. Moreover, T6SS-b was also expressed in the presence of germinated seedlings and in *P. vulgaris* and *Mimosa pudica* root nodules. Phenotypical analysis indicated a role of T6SS-b in motility, suggesting that this cluster may play a role in the early step of plant infection.

## Materials and Methods

### Bacterial Strains, Media, and Cultivation

The strains, plasmids and primers used in this study are listed in the Supplementary Table 1. All *Paraburkholderia* strains were grown in Luria-Bertani (LB) medium without salt at 28°C and 180 rpm (Liu et al., 2020). All other strains were grown in LB medium (Miller, 1972). If needed, the media were supplemented with the appropriate concentrations of antibiotics: chloramphenicol (Cm, 20 μg/ml for *Escherichia coli* and 80 μg/ml for *P. phymatum*), kanamycin (Km, 25 μg/ml for *E. coli* and 50 μg/ml for *P. phymatum*), tetracycline (Tc, 15 μg/ml for *E. coli* and 30 μg/ml for *P. phymatum*), trimethoprim (Trp, 50 μg/ml for *E. coli* and 100 μg/ml for *P. phymatum*). For the induction of the promotors fusions in AB minimal medium (Clark and Maaløe, 1967), the bacteria were grown with different carbon source: 10 mM citrate (ABC), 10 mM glucose (ABG), 12.5 mM glutamate (ABGlu), 15 mM succinate (ABS)/fumarate (ABF)/malate (ABM)/aspartate (ABA). The concentration of the carbon source was adjusted to the number of C-atoms in the molecule. For the plant infection tests, the cultures were washed with AB minimal medium without nitrogen [(A)B medium] (Liu et al., 2020).

### Construction of GFP Reporter Strains and Mutant Strains

The two promotor fusions p5978 (upstream of *tssB* in T6SS-b) and p6115 (upstream of *tssH* in T6SS-3) were constructed using the vector pPROBE-NT as previously described (Lardi et al., 2020). The promotors of Bphy_6116 (gene coding for a hypothetical protein in the second half of T6SS-3), Bphy_7722 (*nodB*), were PCR amplified by using *phymatum* STM815 genomic DNA (gDNA) (Moulin et al., 2014) with primer pairs: p6115_EcoRI_For and p6115_SalI_Rev, p7722_Sal1_For and p7722_EcoR1_Rev, respectively. The amplified fragments were digested and cloned into the vector pPROBE-NT in front of the gene coding for a green fluorescent protein (GFP) (Miller et al., 2000). The cloned sequences were confirmed by sequencing at Microsynth (Balgach, St. Gallen, Switzerland). All constructed plasmids were transferred into *P. phymatum* STM815 by triparental mating. Deletion mutants of T6SSs were constructed by cloning two pieces of DNA sequence flanking the region to be deleted, together with an antibiotic resistance gene in the middle, into a suicide plasmid. The plasmid was then transconjugated into *P. phymatum* STM815 wild-type and plated on selective medium. To construct *P. phymatum* ΔT6SS-b, Bphy_5978 (*tssB*) and Bphy_5979 (*tssC*), which builds the sheath of the T6SS-b, were deleted. The primers Bphy_5978_up_F_NotI and Bphy_5978_up_R_MfeI were used to amplify the upstream fragment and the primers Bphy_5979_dn_F_NdeI and Bphy_5979_dn_R_NotI for the downstream fragment. In between the two fragments, was cloned a trimethoprim resistance cassette *dhfr* with a transcription terminator which was amplified from plasmid p34E-TpTer (Shastri et al., 2017) with the primers Trim_stop_F_NdeI and Trim_stop_R_NdeI. The resulting sequence was then cloned into pSHAFT resulting in pSHAFT::ΔT6SS-b plasmid and the correct construct was confirmed by sequencing. After transconjugation, clones that were chloramphenicol sensitive and trimethoprim resistant were selected as the deletion mutant ΔT6SS-b. In the same way, two ΔT6SS-3 deletion mutants were constructed, where the genes coding for the sheath tssBC (Bphy_6113 and Bphy_6114) were replaced with a trimethoprim cassette (*dhfr*) or a chloramphenicol cassette (*catA2*) cloned from plasmid pSHAFT with the primers catA1_F_EcoR1 and catA2_R_NdeI. The Bphy_6113 upstream fragment was amplified with the primers Bphy_6114_up_F_XbaI and Bphy_6114_up_R_MfeI. The Bphy_6114 downstream fragment was amplified from the gDNA with the primers Bphy_6113_dn_F_NdeI and Bphy_6113_dn_R_XbaI. The ligated inserts were cloned into suicide plasmids pSHAFT and pEX18-Tc, respectively, resulting in constructs pSHAFT::ΔT6SS-3 and pEX18-Tc::ΔT6SS-3. The deletion mutant STM815-ΔT6SS-3 (*dhfr*) was chloramphenicol sensitive and trimethoprim resistant, mutant STM815-ΔT6SS-3 (*catA2*) was tetracycline sensitive and chloramphenicol resistant. To construct a ΔΔ*T6SS* mutant, the plasmid pEX18-Tc::ΔT6SS-3 (*dhfr*) was transferred by triparental mating in *P. phymatum* ΔT6SS-b. The resulting mutant strain was named *P. phymatum* STM815 ΔΔT6SS.

### Expression Analysis

The GFP expression of the promoter reporter strains that were grown in liquid cultures was measured by a plate reader (Tecan Infinite M200 Pro, Tecan Trading AG, Switzerland) with excitation at 488 nm and emission at 520 nm, recording fluorescence in relative fluorescence units (RFU) and the cell density (OD_600_). The GFP expression of bacteria in solid media were visualized by a digital camera (Infinity 3 camera, Lumenera) with GFP filter. The different T6SS reporter constructs (empty pPROBE, p5978, p6115, and p6116) were grown with different carbon sources in 96-well plates (Falcon, Corning, United States) at 30°C for 48 h. Two biological replicates of each strain were tested. To observe the temperature dependent GFP expression of each promoter reporter, bacteria were grown in 50 ml LB-NaCl in 250 ml Erlenmeyer flasks (starting OD_600_ = 0.05) at 180 rpm at different temperatures (20, 28, and 37°C). To visualize the GFP expression responding to the roots of bean germinated seedlings, a suspension of the *P. phymatum* reporter strains (pPROBE, p5978, p6115, p6116, and p7722 at a final OD of OD_600_ = 0.05) was mixed with melted soft agar (0.8%) ABS medium. A germinated bean was placed in the middle of the plate with root tip stabbed into the soft agar medium. After 3 days of incubation at 30°C, the primary roots were inspected under a Leica M165 FC fluorescent stereo microscope, and their relative fluorescent signal acquired through a GFP2 filter set (480/40 nm excitation, 520/10 nm emission) at constant focal distance and acquisition settings (7.3× magnification, 1 s exposure, 1.5× gain). The green channel of resulting RGB images was separated in FiJi and the mean gray signal, 1 mm around each root apex was quantified. The expression of GFP in the nodules of bean and mimosa inoculated with the *P. phymatum* reporter strains (WT-pPROBE, WT-pPROBE-p5978, WT-pPROBE-p6115, WT-pPROBE-p6116) was quantified by using a Leica SPE DM5500Q confocal LASER scanning microscope with an ACS APO 40.0× 1.15 oil objective and the images were analyzed with Fiji (Schindelin et al., 2012). In short, fresh nodule transversal sections were imaged throughout the sample at constant acquisition settings in serial 1 μm optical z-sections from the section plan until the GFP signal was lost. Intact colonized cells were extracted from maximum z-stack projections of the fluorescence channel using the FiJi manual selection tool. Mean gray values were measured for the given number of cells from three discrete plants; 1–2 nodules per plant were examined.

### Phenotypic Analysis

The swimming motility assay was carried out as previously described (Lardi et al., 2020) in a different medium, which was LB plates without salt (0.2% agar). EPS production assay was done as previously described (Liu et al., 2020). Images were taken after 72 h incubation at 28°C. To compare the resistance to H_2_O_2_ and antibiotics of wild-type and mutant strains, soft agar plates were inoculated at a bacterial concentration of OD_600_ = 0.05 in ABS medium. The antibiotic discs (Becton, Dickinson and Company, REF: 231264, 231344, 231299, and 231301) and discs containing 10M, 5M, 1M H_2_O_2_, and H_2_O were placed on the plate and images were taken after 24 h incubation.

### Plant Infection Test

The bean seeds (*P. vulgaris*, cv. Negro Jamapa) and mimosa seeds (*M. pudica*) were surface sterilized as previously described (Talbi et al., 2010; Mishra et al., 2012). Seeds were germinated on 0.8% agarose plates at 28°C. Germinated seeds were planted into yogurt-jars filled with sterile vermiculite (VTT-Group, Muttenz, Switzerland) and Jensen medium (Hahn and Hennecke, 1984). One ml of bacterial cells at an OD_600_ = 0.025 (10^7^ cells pro ml) were inoculated onto each seed and the plants were incubated for 21 (bean) or 28 (mimosa) days in the green house (25°C during day, 22°C at night, 60% humidity, 16 h light per day). The colony forming units (CFU) of each inoculum was determined on LB-NaCl plates. The plants were watered twice a week with autoclaved deionized H_2_O. Symbiotic properties (nodule number, nodule dry weight, and nitrogenase activity) were determined as previously described (Göttfert et al., 1990; Lardi et al., 2017, 2018). Root attachment assay was done as previously described using about 10^7^ cells and a 2-days old germinated seed (Liu et al., 2020).

### Bioinformatics and Statistical Analysis

**FIGURE 1:**
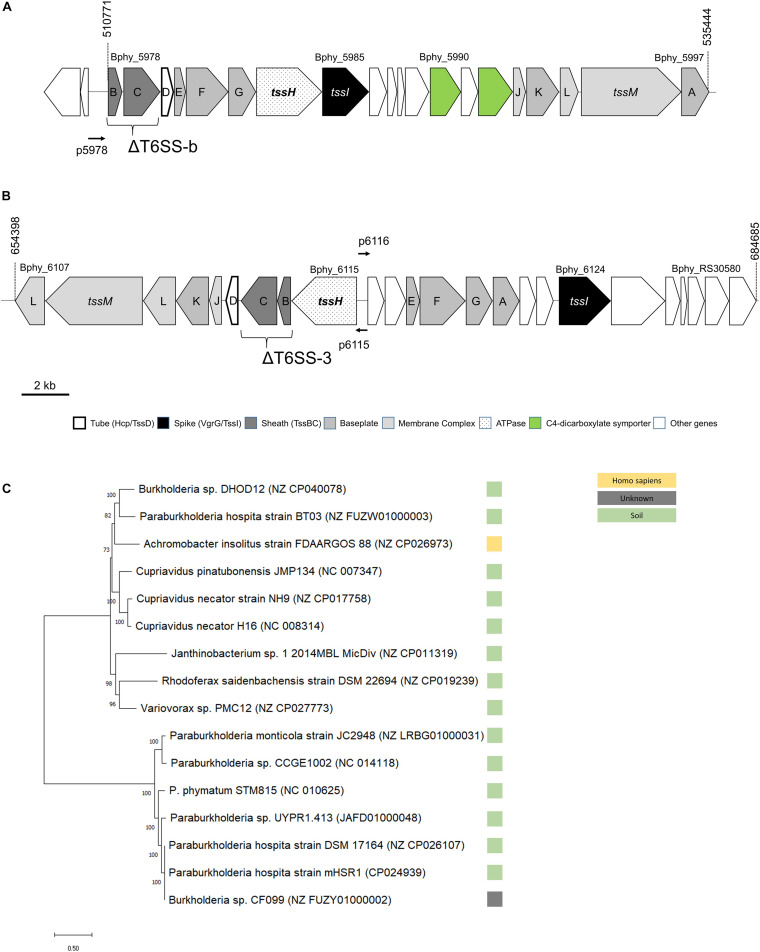
Physical map of the two T6SS loci, **(A)** T6SS-b, **(B)** T6SS-3, and **(C)** unrooted maximum likelihood phylogenetic tree for T6SS-b. In **(A,B)**, numbers on top refer to nucleotide positions according to NCBI. The *tssBC* genes mutated to obtain the T6SS deletion (Δ) mutants are shown. TheT6SS promoters cloned in vector pPROBE are also displayed with a horizontal thin arrow (p5978 with upstream region of Bphy_5978, p6115 with the promoter of Bphy_6115, and p6116 contains the promoter region of Bphy_6116). The phylogenetic tree of T6SS-b **(C)** is based on the DNA sequence as described in section “Materials and Methods”. The reference sequence accession numbers of the NCBI database is shown in brackets and the bootstrap values are shown left of the respective branches. The bar at the bottom left indicates the distance.

To find homologs in other bacteria, the *P. phymatum* T6SS-b and T6SS-3 clusters were searched with blastn 2.9.0+ (Camacho et al., 2009) against the NCBI nt database^1^ in megablast mode using default parameters, a max_target_seq value of 100 and an e-value threshold of 1e-10. For T6SS-b, the nucleotide sequence of the whole cluster (Bphy_5978 to Bphy_5997, NC_010625.1:510771–535444) was searched and sequences of the retrieved homologous clusters were searched again to identify additional, more distantly related clusters. Since the T6SS-3 cluster consisted of two operons pointing in opposite directions (Figure 1B), the sequence of each operon (NC_010625.1:654424–668524 and NC_010625.1:668949–683600) was blasted separately. For strains identified in both Blast searches, the genomic positions of the two operons were visualized in R and strains where matches of both operons co-localized were selected. The identified homologous T6SS-b and T6SS-3 cluster sequences were manually assessed with CLC Genomics Workbench v11.0 (QIAGEN CLC bio, Aarhus, Denmark) to verify that they contained the same core genes as *P. phymatum* and in the same order. ClustalX2 (Larkin et al., 2007) was used to align the manually verified cluster sequences (T6SS-b: *tssB – tssA;* T6SS-3: *tssL* – *tssH*, and Bhy_6116 *– tssA*) and MEGA X was used to construct a maximum likelihood phylogenetic tree with a Tamura-Nei parameter (discrete gamma distribution, five rate categories, bootstrap = 100) (Tamura and Nei, 1993; Kumar et al., 2018). Putative operons were predicted with OperonBD^2^. For the statistical analysis, ANOVA with Tukey’s multiple comparison was performed using GraphPad Prism 7.0 (^∗^*p* ≤ 0.05, ^∗∗^*p* ≤ 0.01, ^∗∗∗^*p* ≤ 0.001, ^****^p ≤ 0.0001).

## Results

### Genomic Analysis of the Two T6SSs Clusters in *P. phymatum* STM815

Two T6SSs clusters have been identified in the *P. phymatum* STM815 genome (Moulin et al., 2014), which consists of two chromosomes and two megaplasmids, with one of them (pBPHY02) containing the symbiotic genes. Both clusters, T6SS-b and T6SS-3, contain all 13 core genes required for the assembly of a fully functional T6SS complex and are located on plasmid pBPHY01 (Figures 1A,B; de Campos et al., 2017). According to the SecReT6 database, these two clusters were assigned to the T6SS-families i4a (T6SS-b) and 3 (T6SS-3) (Barret et al., 2013; Li et al., 2015). While cluster T6SS-b is predicted to be organized as a single operon, T6SS-3 is arranged in two operons facing in opposite direction, one (from Bphy_6107 to 6115) containing *tssHBCDIKLMI* and the other (from Bphy_6116 to Bphy_RS30580) carrying *tssEFGA* and *tssI* (Figure 1B). Each cluster also contains a *vgrG* gene, which codes for a VgrG. Downstream of *vgrG* (Bphy_5985) in the T6SS-b cluster, we found eight genes coding for putative proteins: an YwqK family antitoxin (Bphy_5986), a PAAR-like protein (Bphy_5987), a hypothetical protein (HP) (Bphy_5988), an amino acid ABC transporter (Bphy_5989), a sodium:dicarboxylate symporter (Bphy_5990), an aspartate/glutamate racemase (Bphy_5991) and a second sodium:dicarboxylate symporter (Bphy_5992) (Figure 1A). The presence of two sodium:dicarboxylate symporters in a rhizobial T6SS is intriguing and may suggest a role of T6SS-b during symbiosis, where dicarboxylates are an important source of energy provided by the legume. However, a global search for dicarboxylate transporters in the *P. phymatum* genome revealed the presence of three additional transporters on plasmid pBPHY01 and two genes coding for C4-dicarboxylate transporters similar to DctA encoded on chromosome 1 (Bphy_0225 and Bphy_2596). An alignment of the protein sequences of these different dicarboxylate symporters suggests that the symporters located in T6SS-b likely transport a different substrate compared to that of the known DctA transporters (Supplementary Figure 1; Yurgel and Kahn, 2004). Downstream of *vgrG* (Bphy_6124) located in the T6SS-3 cluster, five genes potentially coding for effectors were identified: two M23 family metallopeptidases (Bphy_6125 and Bphy_RS30575), Bphy_6126 and Bphy_6128 both coding for a HP and Bphy_6127 coding for a PAAR-like protein (Figure 1B). As in many other bacteria, in *P. phymatum*, *vgrG* genes are also found outside of the T6SS-b and T6SS-3 clusters, often containing effector and immunity genes (Santos et al., 2019). *P. phymatum* STM815 contains six orphan *vgrG* copies outside of the T6SS clusters distributed over the genome (Bphy_0023, Bphy_1932, Bphy_3640, Bphy_5197, Bphy_5744, and Bphy_7022). We next looked for the presence of similar T6SS clusters in other strains by blasting the DNA sequence of either the entire cluster for T66S-b, i.e., from Bphy_5978 (*tssB*) to Bphy_5997 (*tssA*), and of the two operons of T6SS-3, i.e., from Bphy_6107 (*tssL*) to Bphy_5107 (*tssH*) and Bphy_6116 to Bphy_RS30575 against the NCBI nt database^3^ (see section “Materials and Methods”). Fifteen strains were found to contain a T6SS-b cluster similar to that of *P. phymatum* STM815. These strains exclusively represent beneficial bacteria occurring in the soil; they include six *Paraburkholderia* strains (*Paraburkholderia monticola* JC2948, *Paraburkholderia atlantica* CCGE1002, *Paraburkholderia* sp. UYPR1.413, *Paraburkholderia hospita* mHSR1 DSM17164 and BT03, two *Burkholderia* strains (*Burkholderia* sp. DHOD12 and sp. CF099), three *Cupriavidus* strains (*Cupriavidus pinatubonensis* JMP134, *Cupriavidus necator* H16 and NH9), *Achromobacter insolitus* FDAARGOS 88, *Variovorax* sp. PMC12, and *Rhodoferax saidenbachensis* DSM22694. One strain (*Janthinobacterium* sp. 1-2014MBL) had three additional genes downstream of *vgrG*, one coding for a GIY-YIG nuclease family protein and two encoding HPs. Moreover, the T6SS-b cluster in *Janthinobacterium* sp. 1-2014MBL did not contain the racemase encoding gene between the two sodium:dicarboxylate symporters. A phylogenetic analysis based on the DNA sequence of the clusters grouped the T6SS-b clusters of these bacteria in two clades (Figure 1C). In contrast, 44 strains were identified (see section “Materials and Methods”) that harbored a cluster similar to T6SS-3 (from Bphy_6107 to Bphy_RS30580), which all belong to different *B. mallei* strains or *B. pseudomallei* strains (Supplementary Figure 2). In order to investigate the expression levels of the T6SSs under different environmental conditions, for each operon, a promotor fusion to the reporter gene *gfp* was constructed (p5978 for T6SS-b, p6115 and p6116 for T6SS-3; Figures 1A,B). While the promoter in p6115 drives expression of the T6SS structural genes, p6116 is the promoter of the operon containing *tssEF* and *vgrG* (*tssI*) together with potential effector genes.

### T6SS-b Is Highly Expressed in Presence of Citrate and T6SS-3 Expression Is Maximal in the Stationary Phase When Succinate Is Used as Carbon Source

**FIGURE 2:**
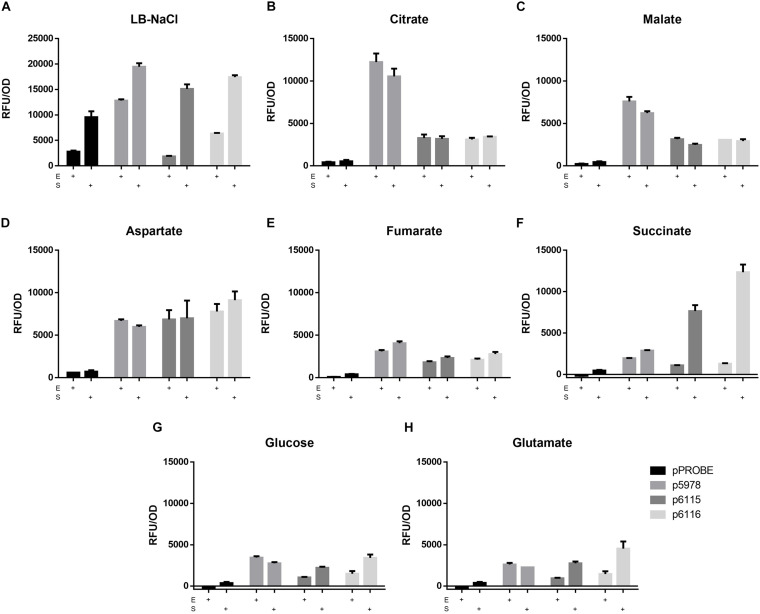
The expression of the promotor fusions (p5978 for T6SS-b and p6115 and p6116 for T6SS-3) in LB-NaCl **(A)** and minimal media [**(B)** citrate, **(C)** malate, **(D)** aspartate, **(E)** fumarate, **(F)** succinate, **(G)** glucose, **(H)** glutamate] with different carbon sources. The expression (GFP) and the growth (OD_600_) were recorded at the end of the exponential phase (E) and the stationary phase (S). A one-way ANOVA with Tukey’s multiple comparisons test was used to analyze the biological duplicates.

As mentioned above, the T6SS-b cluster contains two sodium:dicarboxylate symporters downstream of *vgrG*. To investigate if these transporters (Bphy_5990 and Bphy_5992) are involved in induction of T6SS-b expression, the reporter constructs for T6SS-b (p5978) and T6SS-3 (p6115 and p6116) (Figures 1A,B) were grown in AB minimal media with different C4-dicarboxylates as carbon sources (succinate, malate, fumarate, and aspartate). Additionally, T6SS expression in presence of a C5-dicarboxylate (glutamate), a C6-tricarboxylate (citrate), glucose (C6) as well as in complex medium (LB without salt) was investigated at different time points. The expression levels of the three T6SS reporter constructs at the end of the exponential and in the stationary phase are shown in Figure 2. While in LB without salt all *P*. *phymatum* strains reached the stationary phase after 11 h of growth, the growth rates in minimal media depended on the available carbon source. *P. phymatum* reached the stationary phase after 11, 12, and 14 h with glutamate, succinate, and malate, respectively. With other carbon sources the stationary phase was reached later: glucose (21 h), fumarate (26 h), citrate (36 h), and aspartate (38 h). T6SS-b (p5978) and T6SS-3 (p6115 and p6116) expression was followed for 48 h and in Figure 2 we show the normalized values taken at the end of the exponential phase (E) and in the stationary phase after 48 h (S). In complex medium, T6SS-b showed high levels of expression in both the exponential and the stationary phase, while T6SS-3 was highly expressed only in the stationary phase (Figure 2A). However, the background fluorescence measured in complex medium was very high. When grown in minimal medium, T6SS-b showed the highest expression levels with citrate independent of the growth phase (Figure 2B), a 1.6-fold increase compared to cells grown with malate as carbon source (Figure 2C). The expression of both T6SS-3 operons (p6115 and p6116) in citrate, malate, and fumarate was low and was not induced when the cells grew to the stationary phase. The level of T6SS-b and T6SS-3 expression in a medium containing aspartate was comparable at the end of exponential phase (Figure 2D). When the reporter strains were grown with fumarate, expression of both clusters was low (Figure 2E). While T6SS-b expression remained low in the presence of succinate, expression of T6SS-3 was 9.5-fold upregulated in the stationary phase compared to the end of the exponential phase, showing the highest expression levels for p6116 (Figure 2F). Phase-dependent expression levels were also observed for both T6SS-3 reporters (p6115 and p6116) in minimal medium containing glucose and glutamate (Figures 2G,H, respectively). Conversely, T6SS-b expression remained low in both glucose and glutamate-containing media. Finally, a significant difference in the expression of these two operons (p6115/p6116) was observed in LB-NaCl at the end of the exponential phase. Together, these findings suggested that both T6SS clusters were differentially expressed depending on the available carbon source. T6SS-b showed maximal expression in the presence of citrate independent of the growth phase and highest expression of T6SS-3 was observed in the presence of succinate in the stationary phase.

### Thermoregulation of T6SS Expression

**FIGURE 3:**
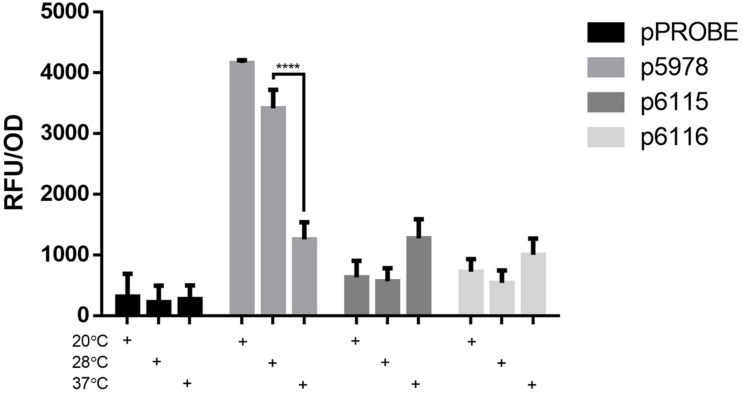
Expression of the T6SS GFP reporter fusion strains (p5978, p6115, and p6116) and a control strain (pPROBE) in LB-NaCl at 180 rpm and 20, 28, or 37°C at the end of exponential phase. The means of biological triplicates are shown with error bars representing the standard deviation. A one-way ANOVA with Tukey’s multiple comparisons test was used to analyze the biological triplicate (^****^p ≤ 0.0001).

Since the entire T6SS-b operon is found in selected other soil bacteria (Figure 1C), while the T6SS-3 cluster is more similar to T6SS from pathogenic *Burkholderia* such as *B. pseudomallei* and *B. mallei* strains (Supplementary Figure 2), we measured expression of both *P. phymatum* T6SS clusters at three different temperatures in LB-NaCl, which mimics soil and host environments. Importantly, the expression was followed over time and the values measured in the exponential phase are shown in Figure 3. Expression of the entire T6SS-b was found to be significantly (*p* ≤ 0.0001) up-regulated when *P. phymatum* was grown at lower temperatures (20 and 28°C). T6SS-b expression increased about 3.3-fold at 20°C compared to 37°C. In contrast, higher T6SS-3 expression levels were determined at 37 vs 28°C (p6115: 2.3-fold and p6116: 1.9-fold; *p* = 0.066 and *p* = 0.42, respectively). Thus, it appears that both systems are subjected to temperature-dependent regulation, which seems to be in line with the different origin of both gene clusters (Figure 1C and Supplementary Figure 2).

### The Presence of a Germinated Seed Induced T6SS-b Expression

**FIGURE 4:**
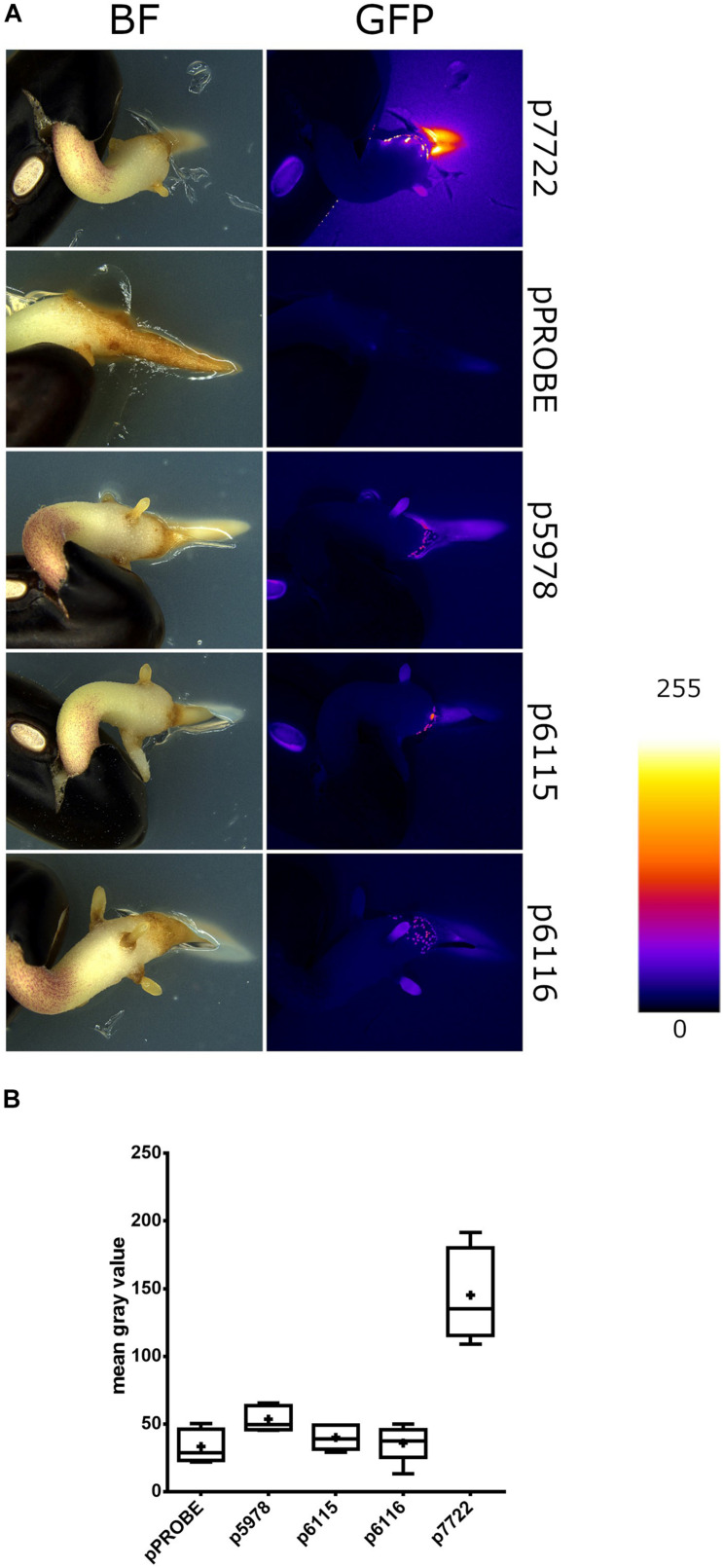
The *gfp* expression of T6SS-b (p5978) and T6SS-3 (p6115 and p6116) clusters on bean roots. **(A)** Representative stereomicrographs. The empty pPROBE vector samples only display plant autofluorescence. All samples were imaged in the same conditions. Fire LUT indicates fluorescent signal intensity. **(B)** Semiquantitative analysis of GFP expression near *P. vulgaris* root tips. Plus signs indicate the arithmetic mean, whiskers minimum to maximum values and black dots outliers according to Tukey’s test. Significant differences between samples were analyzed in a one-way ANOVA with Tukey’s *post hoc* test. Only p7722 values were found statistically different from others (*p*-value < 0.0001).

To assess the expression of *P. phymatum* T6SS during the early steps of the symbiotic process, soft-agar plates containing an OD_600_ = 0.05 of the T6SS reporter constructs p5978, p6115, and p6116 were prepared. The reporter construct p7722, which contains the promotor of Bphy_7722 (*nodB*) fused to *gfp* was used as a positive control. The gene *nodB* is involved in the biosynthesis of the backbone of the NF which is produced in response to flavonoids secreted by the root. Next, a 2 days-old germinated bean seed was added on the plate and the expression was analyzed under a fluorescence stereomicroscope after 3 days of incubation at 28°C. While the T6SS-b cluster (p5978) was expressed, at the tip of the root, both T6SS-3 reporter constructs p6115 and p6116 showed low expression in the presence of the germinated bean seed (Figure 4A). The GFP expression of the reporter constructs was quantified 1 mm around each root apex (Figure 4B). As expected, *nodB* (p7722) was highly expressed displaying a diffused signal around the root, confirming the well-known induction of *nodB* expression by root exudates. Since T6SS-b expression was localized on the root, we suggest that T6SS-b expression is induced by a component present on the tip of the root.

### T6SS-b and T6SS-3 Are Expressed in Plant Root Nodules

**FIGURE 5:**
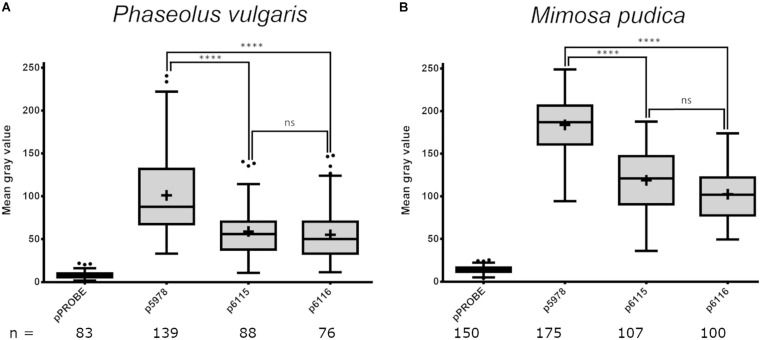
Expression of *P. phymatum* wild-type GFP reporter constructs (pPROBE, p5978, p6115, and p6116) in nodules of *Phaseolus vulgaris* **(A)** and *Mimosa pudica* **(B)**. The pPROBE was used as negative control. Images were acquired with a confocal laser scanning microscope (DM5500Q; Leica) and single cells specific fluorescence quantified with Fiji. Plus signs indicate the arithmetic mean, whiskers minimum to maximum values and black dots outliers according to Tukey’s test. Significant differences between samples were analyzed in a one-way ANOVA with Tukey’s *post hoc* test (^****^p-value < 0.0001, ns, not significant). All T6SS reporter constructs values were found statistically different from pPROBE values.

The expression of both *P. phymatum* T6SSs clusters during symbiosis in root nodules was analyzed using *P. vulgaris* and *M. pudica* as host plants. The plants were infected with the *P. phymatum* T6SS-b (p5978) and T6SS-3 (p6115 and p6116) reporter strains and the root nodules analyzed 21 or 28 dpi. The T6SS-b (p5978) was expressed at a higher level than T6SS-3 (p6115 and p6116) in *P. vulgaris* and *M. pudica* (Figure 5). In the papilionoid plant *P. vulgaris* (Figure 5A and Supplementary Figure 3), T6SS-b and T6SS-3 were expressed at a lower level compared to expression in *M. pudica* nodules (Figure 5B). Both operons of the T6SS-3 cluster (p6115 and p6116) showed a similar expression in *P. vulgaris* and *M. pudica* (Figure 5).

### T6SS-b Is Involved in *P. phymatum* Motility

**FIGURE 6:**
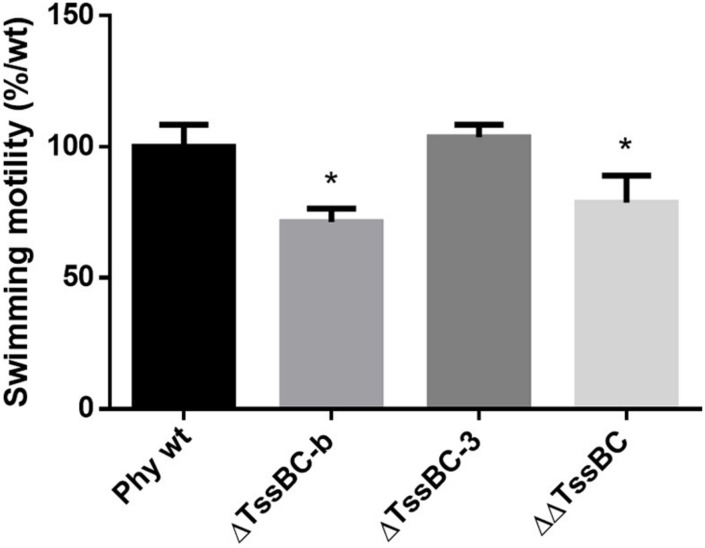
Swimming motility of *P phymatum* wild-type, and the three deletions strains ΔT6SS-b, ΔT6SS-3, and ΔΔT6SS was tested on LB media without salt. The diameter of the halo was measured after 4 days incubation at 28°C. Three biological replicates were analyzed using a one-way ANOVA (*p-value ≤ 0.05), respectively, and bars represent the standard deviation.

To further investigate the role of both T6SSs in *P. phymatum* STM815 during the different steps of the symbiotic interaction with the plants, we deleted the sheath genes (*tssB* and *tssC*) in each T6SS cluster (ΔTssBC-b and ΔTssBC-3) and we constructed a T6SS double mutant strain (ΔΔTssBC). The growth profile of all T6SS mutants in LB without NaCl showed a slightly faster growth of the ΔΔT6SS, while the growth of the ΔT6SS-b and ΔT6SS-3 strains was similar to the growth of the wild-type. We tested phenotypes relevant in different steps of the rhizobium-legume symbiosis such as exopolysaccharide (EPS) production, motility, root attachment, and sensitivity to H_2_O_2_ and antibiotics (Gage and Margolin, 2000; Downie, 2010). While the formation of EPS was not influenced by the T6SS mutant strains (data not shown), the ability to swim was significantly reduced in the ΔT6SS-b and ΔΔT6SS strains (Figure 6). In fact, the ΔT6SS-b and ΔΔT6SS strains showed a 29 and 21% reduced swimming diameter compared to the wild-type strain, respectively. The ability to attach to the roots was tested in a root attachment assay (Liu et al., 2020), revealing no differences between wild-type and mutants (Supplementary Figure 4). Moreover, the three T6SS mutants displayed a similar sensitivity to hydrogen peroxide (H_2_O_2_) and to different antibiotics (gentamycin, kanamycin, and tetracycline) compared to the wild-type (Supplementary Table 2) and the reporter constructs were not activated by the presence of 1 mM H_2_O_2_ (data not shown). Next, the symbiotic properties of ΔT6SS-b, ΔT6SS-3, and ΔΔT6SS strains (nodule numbers, nodule weight, weight per nodule, and normalized nitrogenase activity) were analyzed during symbiosis with *P. vulgaris* and *M. pudica* as host plants and compared with plants infected with *P. phymatum* wild-type (Table 1). While all deletion mutants exhibited no significant differences in the symbiotic properties in either host plant, the ΔT6SS-3 strain showed a 27% lower nodule dry weight in *P. vulgaris* compared to wild-type nodules (Table 1). Further, ΔT6SS-3 infected nodules showed higher nitrogenase activity in *P. vulgaris* and *M. pudica* nodules. However, these tendencies were not statistically significant. In summary, our *in vitro* and *in planta* phenotypic analyses suggested that T6SS-b plays a role in the control of motility and that neither of the T6SSs is required for a functional symbiosis with *P. vulgaris* and *M. pudica*.

**TABLE 1:** The symbiotic properties (nodules per plant, weight per nodule, and nitrogenase activity) of *P. vulgaris* and *M. pudica* nodules infected by *P. phymatum* wild-type, ΔT6SS-b, ΔT6SS-3, and ΔΔT6SS.

Strain	Plant	Number of nodules	Nodule dry weight (mg)	Weight per nodule (mg)	Nitrogen fixation (%/g/min)
**WT**	*P. vulgaris*	109 ± 38	11.96 ± 3.25	0.11 ± 0.02	0.06 ± 0.04
**ΔTssBC-3**	*P. vulgaris*	82 ± 29	8.74 ± 2.37	0.12 ± 0.05	0.13 ± 0.06
**ΔTssBC-b**	*P. vulgaris*	110 ± 11	12.85 ± 1.85	0.12 ± 0.01	0.09 ± 0.04
**ΔΔTssBC**	*P. vulgaris*	103 ± 38	10.28 ± 3.64	0.09 ± 0.03	0.07 ± 0.06
**WT**	*M. pudica*	5 ± 2	0.48 ± 0.13	0.13 ± 0.08	1.34 ± 0.30
**ΔTssBC-3**	*M. pudica*	5 ± 2	0.45 ± 0.09	0.10 ± 0.03	1.43 ± 0.38
**ΔTssBC-b**	*M. pudica*	6 ± 2	0.47 ± 0.19	0.09 ± 0.02	1.25 ± 0.32
**ΔΔTssBC**	*M. pudica*	6 ± 1	0.53 ± 0.05	0.09 ± 0.02	1.29 ± 0.26

*Two biological replicates, consisting of five plants per replicate, were used (*n* = 10) for each strain. Values indicate the average and the standard deviation.*

## Discussion

The role of T6SS during the symbiosis between rhizobia and legumes is still poorly understood. Before T6SS were officially discovered in 2006, the *impJ* gene (later known as *tssK*) of *R. leguminosarum* was shown to be required to extend the host range of this rhizobium (Roest et al., 1997; Bladergroen et al., 2003). Two secreted proteins in this T6SS cluster showed homologies to ribose binding proteins (RbsB) found in *Bacillus subtilis* and *V. cholerae*. These RbsB were shown to be responsible for the blocking of effective nodulation in pea plants (Bladergroen et al., 2003). The exact molecular mechanisms underlying *R. leguminosarum* RbsB function is still unknown. Interestingly, the secretion of these proteins has been shown to be dependent on the environmental temperature and accordingly the effect on the host was stronger when the plants were grown at 24°C compared to 20°C. We report here that the expression of both T6SS in *P. phymatum* is thermoregulated with T6SS-b being expressed at higher levels at 20/28 vs 37°C and T6SS-3 showing the opposite behavior i.e., higher expression at 37°C. While temperatures between 20 and 28°C are typical for the soil environment, temperatures around 37°C are often a signal for the bacterium to be within a mammalian infection host (Cornelis, 2006; Fang et al., 2016). In contrast to most beneficial environmental strains, *P. phymatum* is able to grow well at 37°C, demonstrating that it can adapt to different environmental niches. Until 2014, *P. phymatum* belonged to the highly versatile *Burkholderia* genus, before it was divided into two genera: *Burkholderia* and *Paraburkholderia*, containing pathogenic and non-pathogenic species, respectively (Sawana et al., 2014). This phylogenetic division was used for a first approximation of the pathogenicity of the different strains (Eberl and Vandamme, 2016). In 2016, *Caballeronia* was proposed as a third genus, containing 12 *Burkholderia* and *Paraburkholderia* species (Dobritsa and Samadpour, 2016). *Burkholderia andropogonis* was reclassified in a new fourth genus named *Robbsia* (Lopes-Santos et al., 2017) and two additional genera were recently described (*Mycetohabitans* and *Trinickia*) (Beukes et al., 2017; Estrada-de Los Santos et al., 2018; Mannaa et al., 2018). Interestingly, our study shows that the entire *P. phymatum* T6SS-b cluster also occurs in 15 other environmental strains (Figure 1C), 14 of which were isolated from soil samples. Some of the soil isolates containing T6SS-b are root nodulating bacteria (*Paraburkholderia* sp. UYPR1.413 isolated from *Parapiptadenia rigida* and *Paraburkholderia* sp. CCGE1002 from *Mimosa occidentalis*) or bacteria found in the root endosphere (*P. hospita* BT03 was found in *Populus deltoides*) (Gottel et al., 2011; Ormeño-Orrillo et al., 2012; Taulé et al., 2012). *P. hospita* mHSR1 is a root-associated strain, which induces a systemic resistance against the leaf pathogen *Xanthomonas campestris* in *Brassica oleracea*, and *Variovorax* PMC12 promotes growth of *Solanum lycopersicum* under abiotic stress conditions as well as induces resistance to wilt disease caused by *Ralstonia solanacearum* (Lee et al., 2018; Jeon et al., 2021). Interestingly, downstream of *P. phymatum* VgrG (Bphy_5985) within the T6SS-b cluster, where usually effectors are located, we found two genes coding for potential sodium:dicarboxylate symporters (Bphy_5990 and Bphy_5992; Figure 1A). These transporters are only found in bacteria and belong to the C4-dicarboxylate transporters, a subfamily of the glutamate transporter family that uses C4-dicarboxylates like succinate, fumarate, orotate, aspartate, or malate as substrate (Slotboom et al., 1999). The presence of two putative sodium:dicarboxylate symporters inside a T6SS is intriguing and has to our knowledge not been reported before. Sodium:dicarboxylate symporters are usually located in the inner membrane and may sense the presence of dicarboxylates, i.e., the main carbon source delivered by the plant to the bacteria, leading to an activation of T6SS-b during symbiosis. In *P. aeruginosa*, it has been shown that membrane proteins present in the H1-T6SS cluster are involved in trans-membrane signaling leading to T6SS post-transcriptional activation under optimal environmental conditions (Casabona et al., 2013). To explore the possibility that C4-dicarboxylates activate the expression of *P. phymatum* T6SS-b, we fused the T6SS-b promoter to the reporter *gfp* gene and investigated its expression. Most recently, a study on the opportunistic pathogen *Klebsiella pneumoniae* serotype ST258, pointed to the C4-dicarboxylates itaconate, succinate and fumarate as being inducers of T6SS expression (Wong et al., 2020). While *P. phymatum* T6SS-b was constitutively expressed when cells were grown with the C4-dicarboxylates malate and aspartate as carbon sources, the highest expression of T6SS-b was observed in the presence of the C6 compound citrate. Citrate is known to be exudated by *P. vulgaris* roots under phosphate limited conditions or as part of the aluminum resistance mechanisms of the plant (Shen et al., 2002; Rangel et al., 2010). In case of citrate exudation in response to aluminum resistance, the position of exudation has been shown to be located at the root apices (Rangel et al., 2010). The fact that we found T6SS-b to be expressed to at the root apices (Figure 4), suggests that secretion of citrate from the roots induces T6SS-b expression. A role of T6SS-b during the symbiotic interaction was also suggested by our previous work, which showed that the key symbiotic sigma factor σ^54^ (RpoN) positively controls expression of T6SS-b under nitrogen limiting conditions (Lardi et al., 2020). Although we did not observe any obvious symbiotic phenotype of the T6SS-b mutant, T6SS-b was expressed at the root apices (Figure 4) and in *P. vulgaris* and *M. pudica* root nodules (Figure 5). This suggests that T6SS-b is playing a role during the early symbiotic process. Indeed, a motility test confirmed that a T6SS-b mutant was significantly affected in swimming motility, an important competitive trait for the plant infection process (Figure 6). The involvement of T6SS in motility was already reported in previous studies performed with *P. aeruginosa*, *V. cholera*, and *Pseudomonas fluorescens* (Decoin et al., 2015; Chen et al., 2020; Frederick et al., 2020). A BLAST analysis of the second T6SS cluster in *P. phymatum*, T6SS-3, indicated that the entire cluster is found in the primary pathogens *B. mallei* and *B. pseudomallei* (Supplementary Figure 2). More precisely, *P. phymatum* T6SS-3 is similar to one of the six T6SSs present in the genome of *B. pseudomallei* (T6SS-3; Supplementary Figure 2; Shalom et al., 2007). Although *B. pseudomallei* T6SS-5 has been shown to be a major virulence determinant in animal models (Pilatz et al., 2006; Schell et al., 2007; Schwarz et al., 2014), to our knowledge nothing is known about the function and regulation of T6SS-3. Bioinformatics analysis showed that two putative effector genes found downstream of *vgrG* in this cluster had homology with metallopeptidases that may target cell wall peptidoglycan. The fact that T6SS-3 is more prominently expressed at higher temperatures such as 37°C compared to 28 and 20°C may reflect the environmental condition where this cluster could be used to interact with other prokaryotes or with eukaryotic hosts. Interestingly, in the pathogen *Yersinia pestis* a temperature increase from 28 to 37°C has been shown to induce secretion of proteins required for a functional type III secretion system (T3SS) and for virulence (Cornelis, 2006). Analysis of the expression kinetics of T6SS-b and T6SS-3 indicated that in complex and ABS media, T6SS-b was constitutively expressed, while the T6SS-3 expression increased over time and was maximal at the end of the stationary phase (Figure 2). Constitutive T6SS expression was previously observed in the pathogen *V. cholerae* O37 serogroup strain V52 and suggested to contribute to its fitness in the natural environment (Unterweger et al., 2012). In contrast, regulation of T6SS in *V. cholerae* strain O1 was shown to be cell density dependent with *hcp* expression reacting to environmental factors such as high temperature (37°C) or high osmolarity (340 mM NaCl) (Liu et al., 2008; Ishikawa et al., 2009, 2012). Further stress factors such as acidity, H_2_O_2_ and ethanol also activated expression of T6SS in *Vibrio anguillarum* (Weber et al., 2009). In *P. aeruginosa* T6SS has been shown to react to stress induced by subinhibitory concentrations of antibiotics such as kanamycin or polymyxins (Jones et al., 2013; Vitale et al., 2020). Additionally, *P. aeruginosa* T6SS facilitates the uptake of molybdate and increases its competitiveness (Wang et al., 2021). Our results showed that the expression of *P. phymatum* T6SS was not influenced by the presence of subinhibitory and inhibitory concentrations of several tested antibiotics, at least not at the transcriptional level (data not shown). We also tested if a decrease in pH would affect T6SS expression but could not see a difference when *P. phymatum* was grown at pH 5.5 or 7, suggesting that pH and acidity do not affect T6SS expression (data not shown). Temperature and growth-phase-dependent expression of T6SSs is reminiscent of regulation of virulence factor production in bacterial pathogens, which can involve various regulatory mechanisms such as the stationary phase sigma factor RpoS, different quorum sensing systems or the secondary messenger c-di-GMP (Rossi et al., 2018). *P. phymatum*’s genome contains *rpoS* (Bphy_0962) and the *cepRI* quorum sensing system (Bphy_4439-Bphy_4437), as well as several genes involved in c-di-GMP metabolism. Additional work will be required to unravel the molecular basis of the stationary phase induction of T6SS-3.

Under our experimental settings, we did not identify a role for T6SS-3 in free-living or symbiotic growth conditions. We cannot, however, exclude that in symbiosis with other plants or other host organisms, T6SS-b or T6SS-3 are playing a role or even that the T6SS may have bearing on the exceptional promiscuity of *P. phymatum*. Finally, our study suggests that *P. phymatum* seemingly activates its T6SS copies depending on the environmental niche it is currently occupying, responding to environmental cues such as carbon sources and temperature. We showed that T6SS-b is occurring in other soil-dwelling bacteria and is activated at temperatures found in the soil and in the rhizosphere, which possibly contains citrate, another inducer of T6SS-b expression. In contrast, T6SS-3 is found mostly in pathogenic *Burkholderia* strains and is expressed at highest levels at temperatures usually found in animal hosts (37°C) and in an environment containing succinate or aspartate as a carbon source. The identification of T6SS-b and T6SS-3 effector proteins following induction of each T6SS using the corresponding external clues and analyzing the secretome with proteomics represent important follow-up studies that aim at achieving a better molecular understanding of the respective function(s) of these systems that co-occur in genomes of this versatile group of bacteria.

## Data Availability Statement

The original contributions presented in the study are included in the article/Supplementary Material, further inquiries can be directed to the corresponding author/s.

## Author Contributions

SH and GP conceived and designed the experiments and wrote the manuscript. SH, YL, and AB performed the experiments. SH, BH, AB, CA, LE, and GP analyzed the data. All authors contributed to the article and approved the submitted version.

## Conflict of Interest

The authors declare that the research was conducted in the absence of any commercial or financial relationships that could be construed as a potential conflict of interest.

## Publisher’s Note

All claims expressed in this article are solely those of the authors and do not necessarily represent those of their affiliated organizations, or those of the publisher, the editors and the reviewers. Any product that may be evaluated in this article, or claim that may be made by its manufacturer, is not guaranteed or endorsed by the publisher.

## Footnotes

1 https://ftp.ncbi.nlm.nih.gov/blast/db/, downloaded on January 28, 2020.

2
http://operondb.ccb.jhu.edu/cgi-bin/operons.cgi

3
ftp://ftp.ncbi.nlm.nih.gov/blast/db/

## Supplementary Material

Supplementary Figure 1 Alignment of all C4-dicarboxylate transporter found in *P. phymatum* which are similar to Bphy_5990 compared with other C4-dicarboxylate transporter from Yurgel and Kahn (2004). Glutamate transporter family proteins show eight conserved domains and four conserved motifs. Motif A is the most conserved motif and is hypothesized to correspond to the substrate-binding site.

Supplementary Figure 2 **(A)** The maximum likelihood phylogenetic tree of T6SS-3 was constructed as described in methods based on the DNA sequences of the identified clusters. The reference sequence accession numbers of the NCBI database is shown in brackets and the bootstrap values are shown left of the respective branches. The bar at the bottom left indicates the distance. **(B)** Comparison of the gene arrangement of the two T6SS-3 clusters in *P. phymatum* STM815 and *B. pseudomallei* K96243. NC_006351 from the NCBI database, visualized with CLC Genomics Workbench 11.0.

Supplementary Figure 3 Expression of *P. phymatum* wild-type GFP reporter constructs (pPROBE, p5978, p6115, and p6116) in nodules of *Phaseolus vulgaris*. The pPROBE was used as negative controls. Images were taken with a confocal laser scanning microscope (DM5500Q; Leica). LUT Fire was used to color-code the expression.

Supplementary Figure 4 Root attachment assay with wild-type and T6SS mutants. No significant difference in the root attachment ability was observed between the strains. Three independent experiments were performed with nine biological replicates per strain (*n* = 9). A one way-ANOVA showed no significant difference between the strains. Error bars indicate the standard deviation.
