# Supplementary figures and images for: Differential Expression of Paraburkholderia phymatum Type VI Secretion Systems (T6SS) Suggests a Role of T6SS-b in Early Symbiotic Interaction
Source: Front Plant Sci. 2021 Jul 28;12:699590. doi: 10.3389/fpls.2021.699590 (PMC8356804; doi:10.3389/fpls.2021.699590)

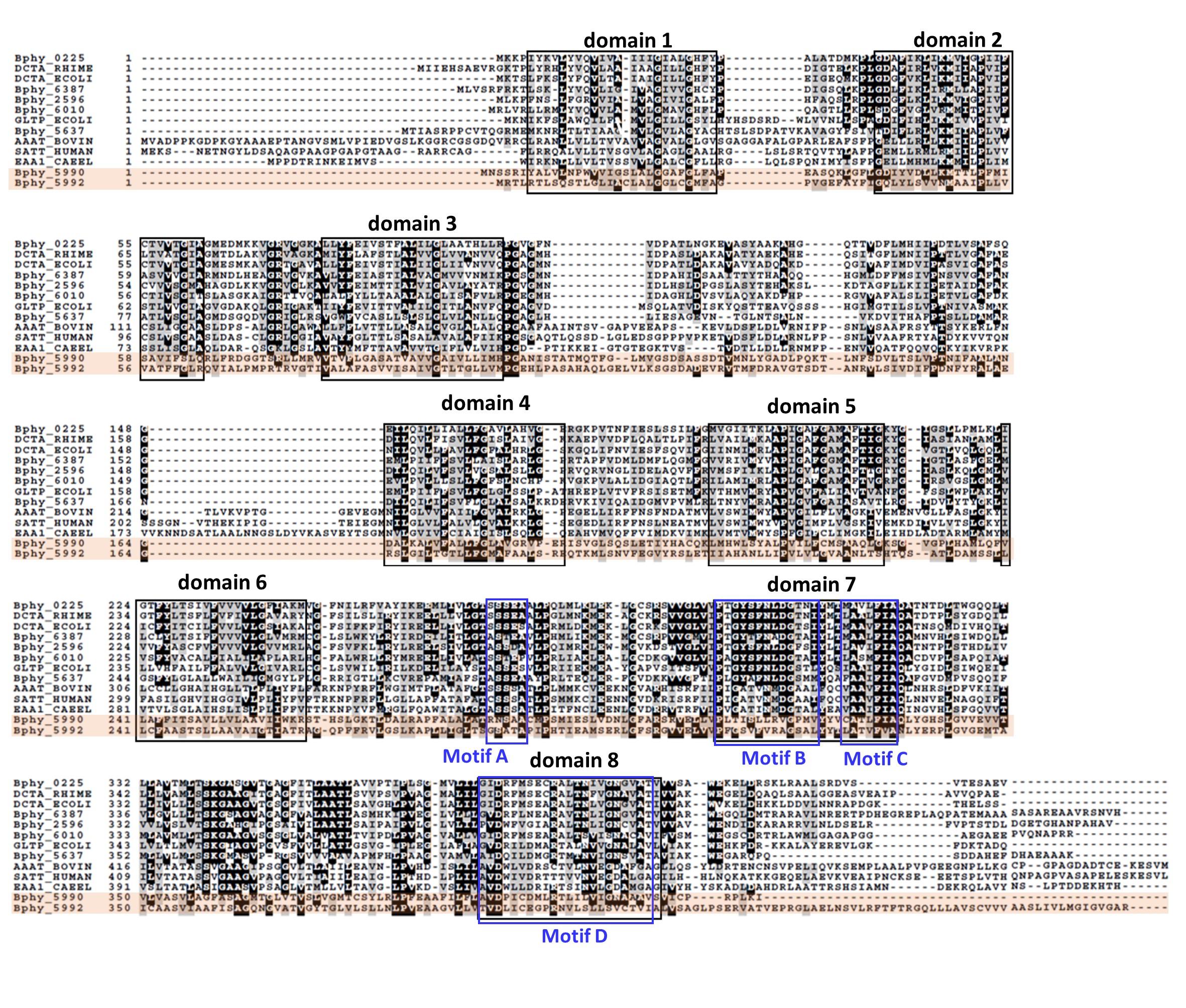

Supplement: Supplementary Figure 1 — Alignment of all C4-dicarboxylate transporter found in P. phymatum which are similar to Bphy_5990 compared with other C4-dicarboxylate transporter from Yurgel and Kahn (2004). Glutamate transporter family proteins show eight conserved domains and four conserved motifs. Motif A is the most conserved motif and is hypothesized to correspond to the substrate-binding site. [file Image_1.JPEG]

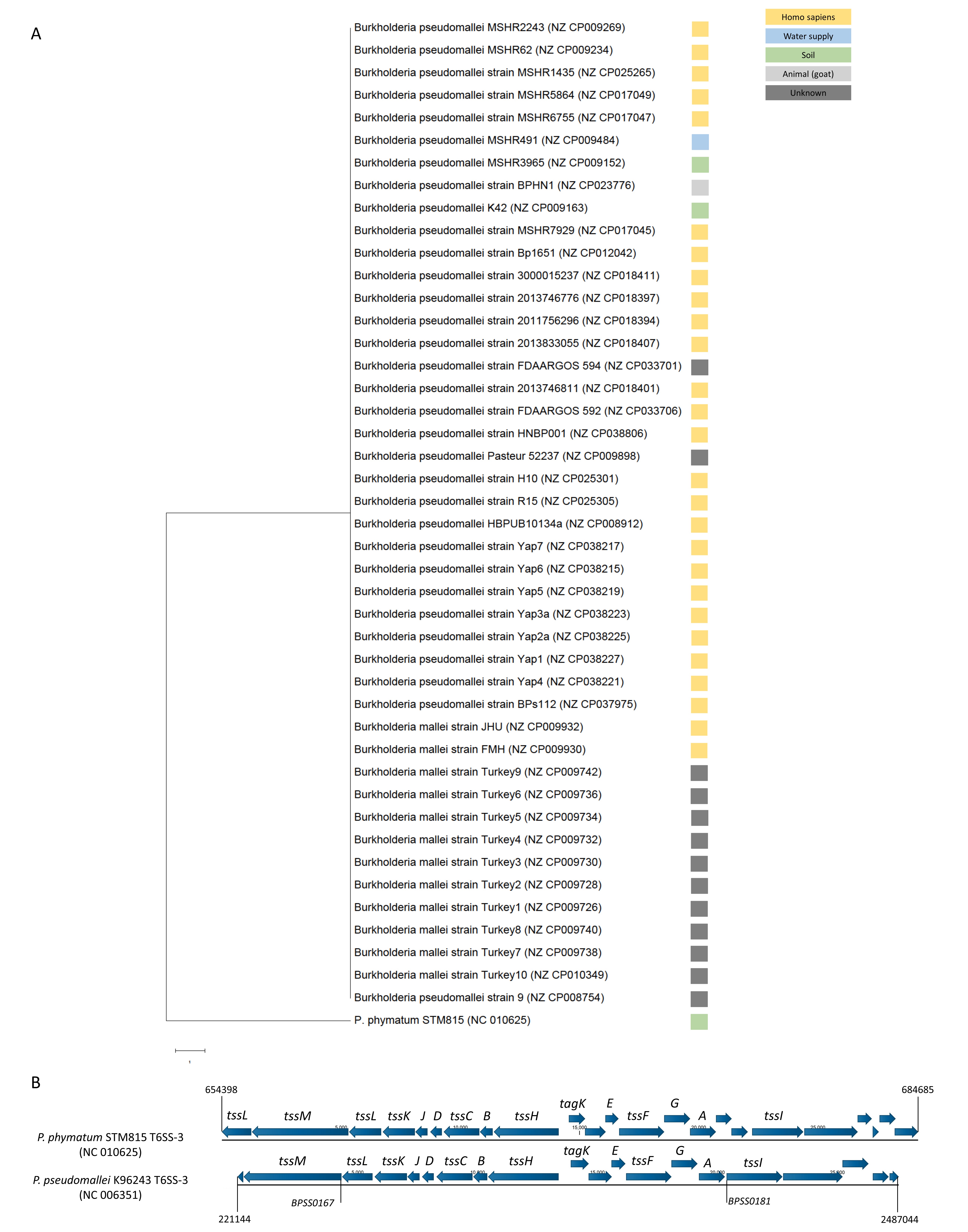

Supplement: Supplementary Figure 2 — (A) The maximum likelihood phylogenetic tree of T6SS-3 was constructed as described in methods based on the DNA sequences of the identified clusters. The reference sequence accession numbers of the NCBI database is shown in brackets and the bootstrap values are shown left of the respective branches. The bar at the bottom left indicates the distance. (B) Comparison of the gene arrangement of the two T6SS-3 clusters in P. phymatum STM815 and B. pseudomallei K96243. NC_006351 from the NCBI database, visualized with CLC Genomics Workbench 11.0. [file Image_2.JPEG]

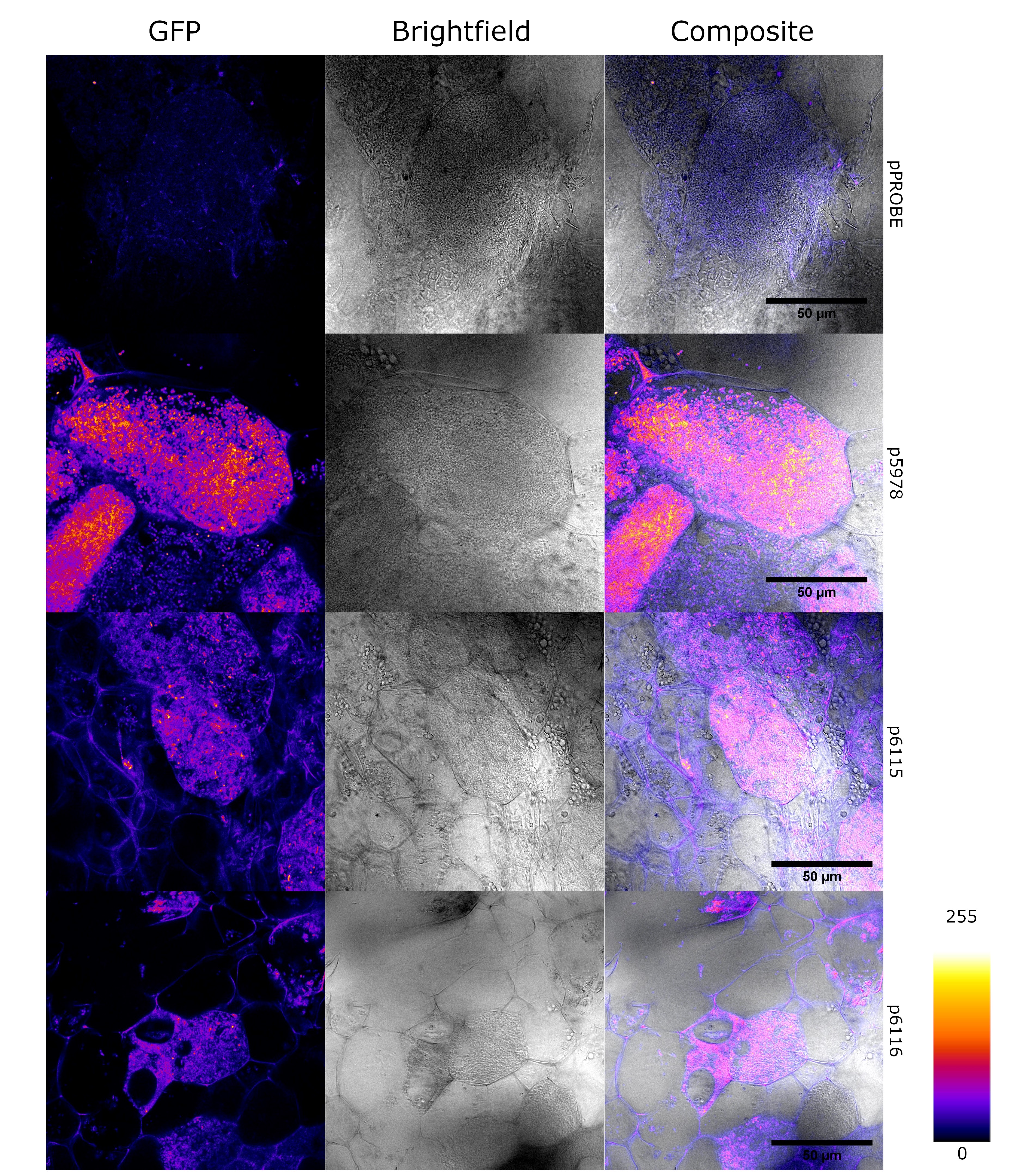

Supplement: Supplementary Figure 3 — Expression of P. phymatum wild-type GFP reporter constructs (pPROBE, p5978, p6115, and p6116) in nodules of Phaseolus vulgaris. The pPROBE was used as negative controls. Images were taken with a confocal laser scanning microscope (DM5500Q; Leica). LUT Fire was used to color-code the expression. [file Image_3.jpg]

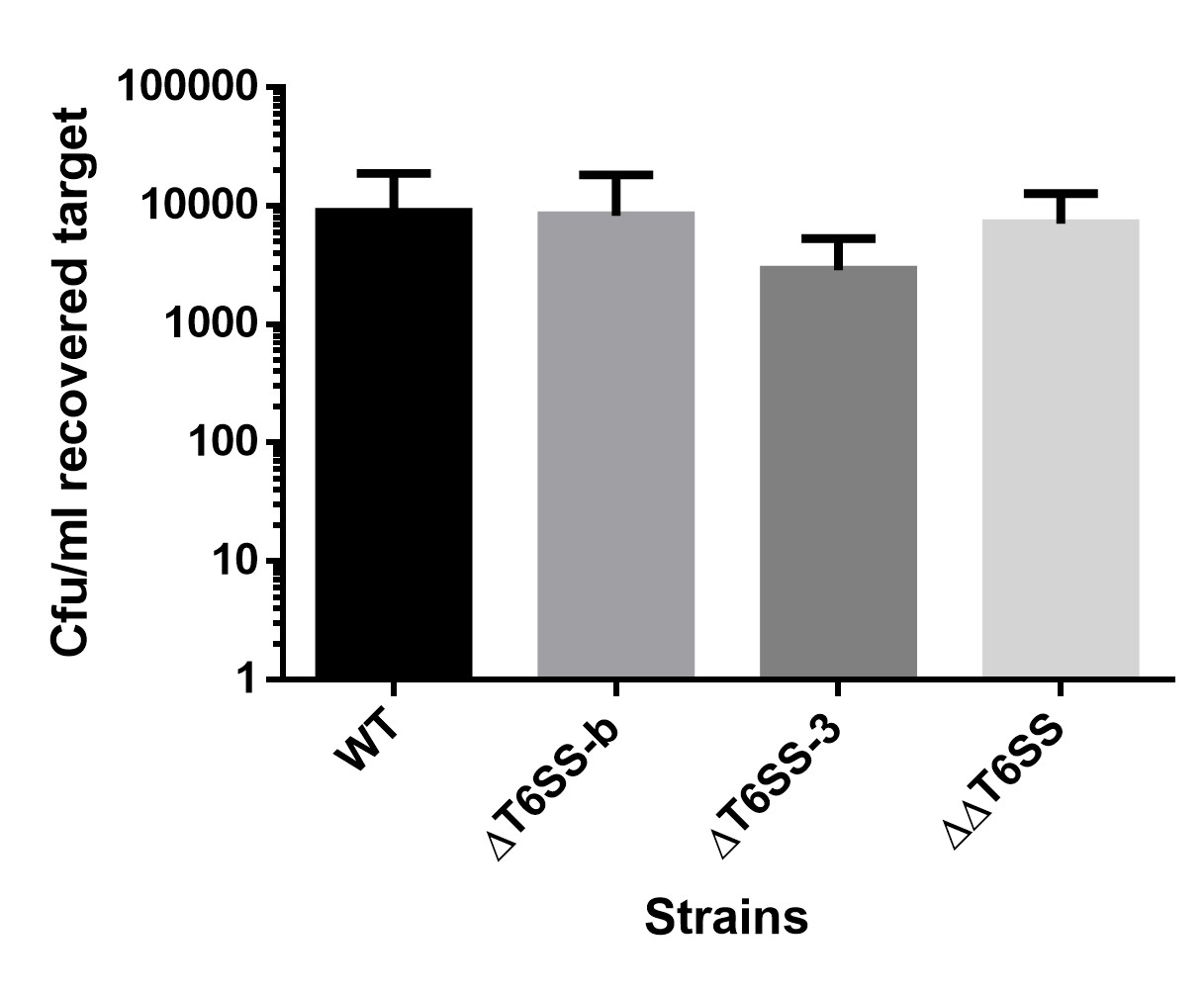

Supplement: Supplementary Figure 4 — Root attachment assay with wild-type and T6SS mutants. No significant difference in the root attachment ability was observed between the strains. Three independent experiments were performed with nine biological replicates per strain (n = 9). A one way-ANOVA showed no significant difference between the strains. Error bars indicate the standard deviation. [file Image_4.jpg]
