# Supplementary material for: Differential Expression of Paraburkholderia phymatum Type VI Secretion Systems (T6SS) Suggests a Role of T6SS-b in Early Symbiotic Interaction
Source: Front Plant Sci. 2021 Jul 28;12:699590. doi: 10.3389/fpls.2021.699590 (PMC8356804; doi:10.3389/fpls.2021.699590)
Supplement: Supplementary file 5 [file Data_Sheet_1.docx]

# Supplementary Material

**Supplementary** Table 1: Used strains, plasmids and oligonucleotides.

| Strains | Description | | Reference |
| --- | --- | --- | --- |
| *P. phymatum* STM815 |  | |  |
| WT | Wild type | | (Moulin et al., 2001) |
| WT-pPROBE | pPROBE-NT without promotor; Km^R^ | | (Liu et al., 2020) |
| WT-pPROBE-p5978 | pPROBE-NT::pBphy_5978; Km^R^ | | (Lardi et al., 2020) |
| WT-pPROBE-p6115 | pPROBE-NT::pBphy_6115; Km^R^ | | (Lardi et al., 2020) |
| WT-pPROBE-p6116 | pPROBE-NT::pBphy_6116; Km^R^ | | This study |
| WT-pPROBE-p7722 | pPROBE-NT::pBphy_7722; Km^R^ | | This study |
| STM815-ΔT6SS-b | STM815, *tssB-tssC* deletion mutant, Position: 663709-665754; Tmp^R^ | | This study |
| STM815-ΔT6SS-3 (*dhfr*) | STM815, *tssB-tssC* deletion mutant, Position: 1917109-1919120; Tmp^R^ | | This study |
| STM815-ΔT6SS-3 (*catA2*) | STM815, *tssB-tssC* deletion mutant, Position: 1917109-1919120; Cm^R^ | | This study |
| STM815-ΔΔT6SS | STM815, *tssB-tssC* deletion mutant, Positions: 663709-665754 and 1917109-1919120; Cm^R^, Tmp^R^ | | This study |
| *E. coli* |  | |  |
| c118 λ-pir | Δ*(ara-leu) araD* Δ*lac74 galE galK phoA20 thi1 rpsE rpoB argE(Am)*  *recAI* λ *pir;* Strep^R^ | | (Herrero et al., 1990) |
| Top10 | Δ*lac*X47 *ara*Δ139Δ(ara-leu) | | Invitrogen ® |
| Plasmids | **Description** | | **Reference** |
| pPROBE-NT | Broad-hoast-range promotor-probe vector; Km^R^ | | (Miller et al., 2000) |
| pPROBE-NT::p5978 | pPROBE with Bphy_5978 promotor region in front of *gfp*; Km^R^ | | (Lardi et al., 2020) |
| pPROBE-NT::p6115 | pPROBE with Bphy_6115 promotor region in front of *gfp*; Km^R^ | | (Lardi et al., 2020) |
| pPROBE-NT::p6116 | pPROBE with Bphy_6116 promotor region in front of *gfp*; Km^R^ | | This study |
| pPROBE-NT::p7722 | pPROBE with Bphy_7722 promotor region in front of *gfp*; Km^R^ | | This study |
| pRK2013 | Helper plasmid; Km^R^ | | (Figurski and Helinski, 1979) |
| pSHAFT | Broad-host-range suicide plasmid; Cm^R^ | | (Shastri et al., 2017) |
| pSHAFT::ΔT6SS-b | pSHAFT2 carrying a 1679 bp fragment for deletion of *Bphy_5978* and *Bphy_5979* | | This study |
| pEX18-Tc | Suicide vector pEX18Tc | | (Biswas and Mettlach, 2019) |
| pEXT18-Tc::ΔT6SS-3 | pEX18-Tc carrying a 1951 bp fragment for deletion of *Bphy_6113* and *Bphy_6114* | | This study |
| pEXT18-Tc::ΔT6SS-3 | pEX18-Tc carrying a 1951 bp fragment for deletion of *Bphy_6113* and *Bphy_6114* | | This study |
| p34E-TpTer | p34E containing dfrB2 gene fused to rrnB, T1T2 terminators (Ap^R^ , Tp^R^) | | (Shastri et al., 2017) |
|  | |  |  |
| Oligonucleotides | | **Sequence** | **Source** |
| p6115_EcoRI_For  p6115_SalI_rev  p7722_Sal1_For  p7722_Sal1_Rev  Bphy_5978_up_F_NotI  Bphy_5978_up_R_MfeI  dhfrFor_EcoRI  dhfrRev_NdeI  Bphy_5979_dn_F_NdeI  Bphy_5979_dn_R_NotI  Bphy_6114_up_F_XbaI  Bphy_6114_up_R_MfeI  Bphy_6113_dn_F_NdeI  Bphy_6113_dn_R_XbaI  catA2_F_EcoRI  catA2_R_NdeI  Trim_stop_F_NdeI  Trim_stop_R_NdeI | | TTTgaattcGCGCGGGTCGCGGGCGAT  TTTgtcgacCGTCATGAGTTTCTTCCA  TTTgtcgacTGGAAGACGTCGCGTGAA  TTTgaattcCGACTATTCATGGTGGTG  GCGCGCggccGCTCGTACGTCCGTCCTTTTTC  CGCGcaattgAAACCTCTCCGGAAAAGCAC  CGCGgaattcCAGTTGACATAAGCCTGTTC  CGCGcatatgTAGGCCACACGTTCAAGTG  CGCGcatatgTGATGTTCCGTCTCGCGC  GCGCgcggccgcCCGACACGTTCTTCGACA  GCGCtctagaCTGACGGCAAGCCTGTGT  CGCGcaattgTGGGGGAAACTCCCGTGA  CGCGcatatgGCCTGATTCCGGCGGATC  GCGCtctagaTGTTGCCCTTGATGTCGTAG  GCGCgaattcTTGACAATTAAGCCCGTATATGG  GCGCcatatgCCGGATACGGTGGCTTAAAT  GCGCcatatgGAGAGTAGGGAACTGCCAG  GCGCcatatgGTAGATATGACGACAGGAAGA | This study  This study  This study  This study  This study  This study  This study  This study  This study  This study  This study  This study  This study  This study  This study  This study  This study  This study |

**Supplementary** Table 2: Resistance to H_2_O_2_ and antibiotics. The diameter of the inhibition zone (cm) was measured in ABS media with *P. phymatum* wild-type, ΔT6SS-b, ΔT6SS-3 and ΔΔT6SS. Three biological replicates were tested for each strain (n = 3). Mean value ± standard deviation are shown.

| Strain | 10M H_2_O_2_ | 5M H_2_O_2_ | 1M H_2_O_2_ | 0M H_2_O_2_ | Gentamycin 10 | Kanamycin 30 | Tetracycline 30 |
| --- | --- | --- | --- | --- | --- | --- | --- |
| WT | 2.34  ± 0.1 | 2.0  ± 0.02 | 1.19  ± 0.04 | 0 | 0.97  ± 0.17 | 0.97  ± 0.21 | 2.86  ± 0.3 |
| ΔTssBC-b | 2.17  ± 0.05 | 1.9  ± 0.05 | 1.1  ± 0.02 | 0 | 1.0  ± 0.07 | 1.12  ± 0.05 | 2.85  ± 0.02 |
| ΔTssBC-3 | 2.47  ± 0.1 | 2.23  ± 0.13 | 1.36  ± 0.17 | 0 | 0.97  ± 0.1 | 1.14  ± 0.15 | 2.97  ± 0.04 |
| ΔΔTssBC | 2.51  ± 0.23 | 2.23  ± 0.19 | 1.47  ± 0.24 | 0 | 0.88  ± 0.02 | 1.03  ± 0.03 | 2.82  ± 0.07 |
